# Predicting Class II MHC-Peptide binding: a kernel based approach using similarity scores

**DOI:** 10.1186/1471-2105-7-501

**Published:** 2006-11-14

**Authors:** Jesper Salomon, Darren R Flower

**Affiliations:** 1 The Jenner Institute, University of Oxford, Compton, Newbury, Berkshire, RG20 7NN, UK

## Abstract

**Background:**

Modelling the interaction between potentially antigenic peptides and Major Histocompatibility Complex (MHC) molecules is a key step in identifying potential T-cell epitopes. For Class II MHC alleles, the binding groove is open at both ends, causing ambiguity in the positional alignment between the groove and peptide, as well as creating uncertainty as to what parts of the peptide interact with the MHC. Moreover, the antigenic peptides have variable lengths, making naive modelling methods difficult to apply. This paper introduces a kernel method that can handle variable length peptides effectively by quantifying similarities between peptide sequences and integrating these into the kernel.

**Results:**

The kernel approach presented here shows increased prediction accuracy with a significantly higher number of true positives and negatives on multiple MHC class II alleles, when testing data sets from MHCPEP [1], MCHBN [2], and MHCBench [3]. Evaluation by cross validation, when segregating binders and non-binders, produced an average of 0.824 A_ROC _for the MHCBench data sets (up from 0.756), and an average of 0.96 A_ROC _for multiple alleles of the MHCPEP database.

**Conclusion:**

The method improves performance over existing state-of-the-art methods of MHC class II peptide binding predictions by using a custom, knowledge-based representation of peptides. Similarity scores, in contrast to a fixed-length, pocket-specific representation of amino acids, provide a flexible and powerful way of modelling MHC binding, and can easily be applied to other dynamic sequence problems.

## Background

Major Histocompatibility Complexes (MHC) bind short peptides derived from antigens and present them on the cell surface for inspection by T-cells. The binding mechanism appears to be the most selective step in the recognition of T-cell epitopes. The molecular mechanisms underlying this selectivity are still debated [4], but a crucial factor is the complementarity between amino acids in the antigen peptide and the MHC binding pocket [5]. Successfully modelling the behaviour exhibited by MHCs can be used to pre-select candidate peptides, which, in turn, can limit the practical work involved and facilitate the search for new vaccines.

MHC alleles are grouped according to their structure. For class I MHC alleles, the binding groove is closed at both ends, making it possible to predict exactly which residues are positioned in the binding groove. For Class II MHC molecules, the binding groove is open at both ends and peptides which bind class II alleles are generally longer than those which bind class I MHCs, typically 9 to 25 residues. Moreover, the grooves of MHC Class II alleles will only accommodate 9 to 11 residues of the target peptide [6]. Thus class II peptides have the potential to bind to the MHC groove in one of several registers (potential alignments between groove and antigenic peptide).

Interaction, within the groove, between MHC and peptide side chains is generally considered the principal determinant of binding affinity [7]. However, for MHC Class II type alleles, a recent study speculates that binding may not be completely deterministic, and that the same peptide can have multiple possible binding cores [8]. Moreover, several studies have shown that the binding core is, indeed, not the only factor; residues outside the binding groove (flanking residues) can also interact with the MHC molecule and influence binding [9-14]. Hence, this creates additional complexity in determining which residues are involved in the interaction, and suggests that a suitable method must include a full-length representation of the peptide.

Numerous methods have been applied to the problem of predicting MHC binding. Prediction of MHC class I binding has been very successful, reporting prediction accuracies of up to 95% (e.g. [15]). Attempts at predicting class II MHC binding show significantly lower accuracies, although many efforts using both traditional and novel approaches have been applied, some demonstrating inspirational progress.

In recent years, efficient pattern recognition methods have been applied to the class II problem, such as Artificial Neural Networks [7,16,17] and Support Vector Machines [18,19]. However, these methods are based on inductive learning and require fixed-size representations to perform attribute-by-attribute comparisons of input variables. A typical approach for such methods is to first estimate (or input) a binding core, and subsequently predict the binding affinity of an unknown peptide based on the estimated core (typically a nonamer). This 2-step process is convenient from a mathematical modelling perspective, because it restricts the prediction task to a fixed-length formulation (9-mers) and thus avoids the problem of handling variable length peptides. The subsequent conversion of the 9-mer amino acid representation into a numerical representation is achieved by using either a binary positional system with 20 inputs per amino acid [17,18,20-22], or by using amino acid properties [23,24]. The results are fixed-length, high-dimensional, input vectors used for training the model (up to 180 dimensions in the case of the binary positional system).

Approaches for solving the dynamic nature of the prediction problem, and which can handle the variability in peptide lengths, have shown promising prediction qualities. Methods include an iterative "meta-search" algorithm [20], an iterative Partial Least Squares method [22], Hidden Markov Models [16,25], an Ant Colony search [26], and a Gibbs sampling algorithm [27]. Some of these novel approaches have produced remarkable results, significantly outperforming conventional approaches.

The method presented here aims to combine the advantages of the two approaches: It utilises an efficient fixed-length discriminative method, but is still able to handle variable length peptides. This is achieved by applying a customised kernel.

Kernel methods have become popular thanks to Support Vector Machines (SVMs), originally introduced by Vapnik [28]. They have been applied to multiple bioinformatical problems, and have shown excellent performance using real-world data sets (see [29] for examples). In its basic form, a single SVM is a binary classifier which learns a decision boundary between two classes (e.g. binders and non-binders) in some input space (e.g. vectors with some amino acid representation). To find a decision boundary between two classes, an SVM attempts to maximise the margin between the classes, and choose a linear separation in a feature space. A function called the *kernel function K *(*x*_*i*_, *x*) is used to project the data from input space to feature space, and if this projection is non-linear it allows for non-linear decision boundaries. The effectiveness of SVMs is due to two factors: a) the principle of maximising margins (structural as opposed to empirical risk minimisation) and b) using the *kernel trick *to extend linear methods so that they can address non-linear problems. Details of the SVM formulation have been described thoroughly in many books and publications (e.g. [30]).

An advantage of kernel methods, which render them particularly suited for problems in computational biology, is the ability to customise the kernel. The kernel can be seen as a distance measure between two samples, e.g. in the case of a linear kernel the Euclidean distance between two samples. A custom kernel can be used to define explicitly a distance measure between two samples, and thus knowledge-based kernels can be designed to process variable length data and convert samples into fixed-length representations needed for direct comparisons. For sequences of proteins, it can be used to define similarity measures between pairs of sequences (proteins, peptide strings, etc). Methods utilising such *direct kernel functions *have lead to significant improvements in performance on classical bioinformatical problems, such as remote homology detection [31,32] and protein classification [33].

In this paper, we present a kernel method based on the direct kernel function of [32], which we have adapted to the problem of predicting MHC binding. The *Local Alignment Kernel *is a kernel quantifying the similarity between a pair of protein sequences by taking into account *all *possible optimal alignment scores between *all *possible sub-sequences.

## Results

Using several sets of data (see Table 1), a method for the prediction of class II epitopes was developed and subsequently optimised. Initially, the effect on accuracy of varying the two parameters of the model was explored; these include a regulatory parameter β and a substitution matrix *S *(·), which are both described in detail below. Tests were then run to compare the performance of this kernel approach with existing prediction methods.

**Table 1:** Overview of data sets

**Name**	**Data set**	**Samples**	**Binders**	**Non-binders**
MHCBN	HLA-DRB1*0101	580	475	105
	HLA-DRB1*0301	369	219	150
MHCBench	Set 1	1017	694	323
	Set 2	673	381	292
	Set 3a	590	373	217
	Set 3b	495	279	216
	Set 4a	646	323	323
	Set 4b	584	292	292
	Set 5a	117	70	47
	Set 5b	85	48	37
MHCPEP	20 sets from MHC alleles	3578	3578	0

Overview of the benchmark data sets. MHCBench Sets 1–5 contain data from the HLA-DRB1*0401 allele. MHCPEP consists of data from numerous alleles, with 18 MHC Class II and a single MHC Class I allele selected.

### Optimising the β-parameter

The kernel is based on similarity scores between pairs of peptides. For each pair, a similarity score is composed of multiple sub-scores based on alignments between pairs of sub-sequences. The model parameter β regulates the relative influence that each sub-score will have on the cumulative score. In turn, it enables adjustment of the importance of sub-optimal alignments.

Experiments were undertaken to evaluate the effect on performance of varying the β-parameter using a simple test set. The MHCBench Set 4b was chosen for this purpose; it contains experimentally verified binders and non-binders of HLA-DRB1*0401. It consists of only natural peptides and an equal number of binders and non-binders (292 of each), which makes it well-suited for model testing.

**Figure 1:**
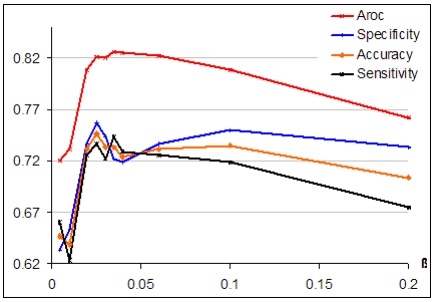
Evaluating performance for varying the β-parameter using 10-fold CV. Graphs are plotted for accuracy (proportion of correct predictions), sensitivity (proportion of false negatives), specificity (proportion of true positives), and A_ROC _(area under receiver operating characteristic curve).

The BLOSUM62 substitution matrix was used for *S *(·). This matrix is generally considered to be a good matrix for modelling evolutionary problems [34]. 10-fold CV was used to evaluate performance, and a rough search for a good β-parameter was undertaken. The effect on performance of varying β can be seen in Figure 1, which shows that the SKM is capable of distinguishing well between binders and non-binders. The best accuracy is 74.7% at β = 0.025. The best performance in terms of A_ROC _is 0.827 at β = 0.035. Generally, the best results for most measures are found for β-values between 0.02 and 0.04. Higher β values of 0.2 to 5.0 were also evaluated, but as β becomes larger performance degrades for all measures.

Bootstrapping using case resampling [35] was performed to analyse the variance in results. 100 repetitions were undertaken, with data set sizes of 584. At a β-value of 0.025, which produced the best accuracy in the tests referred to above, the average bootstrapping accuracy was 73.1% with a standard deviation of 2.2%. This degree of variance was found throughout our experiments.

Interestingly, a low β indicates that the best solution is found when sub-optimal alignments have a large influence, as seen by the mathematical formulation below; when lowering the β-value, the relative contributions from scores of various alignments are evened out. This tendency was observed throughout the remaining experiments, with most "optimal" β-values being below 0.1. Interestingly, the same observation regarding the positive influence of sub-optimal alignments was also reported in Nielsen et al [27] using a very different method.

### Selecting the substitution matrix

A substitution matrix was used in the calculation of the Smith-Waterman score to evaluate similarities between amino acids (see Eq. 0.3). The BLOSUM62 matrix initially used is regarded as a good descriptor for evolutionary problems [34]. However, using an alternative substitution matrix could prove more effective.

The AAIndex database [23] contains a large collection of substitution matrices produced during the last three decades. The matrices are based on numerous different measures, such as physicochemical properties and structural differences. An extensive search among the substitution matrices was conducted. Each substitution matrix was used instead of the BLOSUM62 matrix as above. Due to the scale of the experiment, only a crude search using 5 values of β was evaluated per substitution matrix. From the 83 matrices, the ten best performing substitution matrices with regard to A_ROC _scores were retested with a refined search for the best value of β. The 3 best performing substitution matrices from this experiment are shown in Table 2.

**Table 2:** Results from varying substitution matrices

**AAindex reference**	**Substitution matrix**	**Best β**	**Acc.**	**A**_**ROC**_	**A**_**ROC50**_	**MCC**
HENS920102	BLOSUM62. Matrix based on possible pair-wise substitutions from aligned segments of polypeptides [34]	0.05	0.8049	0.8708	0.532	0.543
BLAJ010101	Matrix built from structural superposition data for identifying potential remote homologues [52]	0.027	0.8207	0.8752	0.540	0.571
DOSZ010104	SM_THREADER_NORM. Amino acid similarity matrices based on force fields (Normalised version) [36]	0.045	**0.8217**	**0.8753**	**0.548**	**0.572**

Evaluating performance for using different scoring matrices using 10-fold CV. The test measurements are same as in previous experiment. Best values are shown in bold.

As can be seen from the table, the three matrices have very similar A_ROC _values. The best performance was produced by a recently developed substitution matrix SM_THREADER_NORM, which is based on molecular mechanics force fields. [36] suggest that force fields can provide more reliable mutation matrices because of the incorporation of natural weighting of different physical contributions. Interestingly, the BLOSUM62 matrix is among the best three matrices out of 83. This suggests that the evolutionary rationale behind BLOSUM62 is also appropriate for MHC peptide similarity or that the chemical similarities underlying protein evolution also underlie peptide selectivity by the MHC. Such conjecture is supported in part by the fact that the SM_THREADER_NORM is also placed in the same family of substitution matrices when assessing the magnitude of distances between matrices [36]. In the following experiments, the SM_THREADER_NORM is used.

### Performance on HLA-DRB1*0101 and HLA-DRB1*0301

Two MHC Class II alleles from the MHCBN database [2] were evaluated. The MHCBN database contains 475 binders and 105 non-binders for HLA-DRB1*0101 and for HLA-DRB1*0301 contains 219 binders and 150 non-binders. Duplicates and peptides with 75% or more Alanines were removed. 5-fold cross-validation was undertaken (5 fold CV used instead of 10-fold CV for comparison with [21]), and a crude optimisation of the β-parameter was performed as described in the methods section. Results are shown in Table 3. The table reports comparison results from two other methods, a linear programming model "LP_top2" [21] and TEPITOPE, a quantitative matrix method [37].

**Table 3:** Results on HLA-DRB1*0101 and HLA-DRB1*0301

**Allele**	**Method**	**Acc.**	**A**_**ROC**_	**A**_**ROC50**_	**MCC**	**A**_**OVER-ROC**_
HLA-DRB1*0101	SKM (β = 0.04)	0.886	**0.912**	**0.804**	0.643	**0.088**
	SKM (β = 0.085)	**0.901**	0.904	0.778	**0.690**	0.096
	LP_top2		0.779			0.221
	TEPITOPE		0.842			0.158

HLA-DRB1*0301	SKM (β = 0.06)	**0.763**	0.823	**0.580**	**0.531**	0.177
	SKM (β = 0.08)	0.757	**0.827**	0.575	0.525	**0.173**
	LP_top2		0.721			0.279
	TEPITOPE		0.585			0.415

Results of 5-fold cross-validation with best results shown in bold. Results from LP_top2 and TEPITOPE are taken from [21]. Measurements are same as previously reported, except for AOVER-ROC, which is the area over the ROC curve. AROC = 1.00 is perfect classification, so AOVER-ROC, 1- AROC, can be seen as an error measure.

The results in Table 3 show that the SKM method performs significantly better than LP_top2 and TEPITOPE. The relative improvements in A_ROC _scores are 8% and 41%, and the improvements in A_OVER-ROC _are 37% to 60%. Other methods have also been evaluated on the data sets. In [25], results on two methods using Hidden Markov models combined with successive state splitting are reported. The best 10-fold CV results were 0.85 (S-HMM1) and 0.89 (S-HMM2), which are close to but still lower than the 0.91 A_ROC _of SKM using 5-fold CV; using 10-fold CV the SKM performance increases to 0.93 A_ROC_.

### Performance on allele HLA-DRB1*0401

MHCBench [3] contains 8 data sets of binders and non-binders for HLA-DRB1*0401. Again, the SKM was evaluated using 10-fold cross validation, and a crude search for optimal values of β was performed for each set. A_ROC _performance from all 8 sets are reported in Table 4, which also includes results of PERUN, a method based on TEPITOPE [38], Neural Networks [7], Gibbs Sampler, a method based on Metropolis sampling [27], and LP_top2, a linear programming method [21].

**Table 4:** Comparison of A_ROC _values on HLA-DRB1*0401 data sets from MHCBench

**Method**	**Set1**	**Set2**	**Set3a**	**Set3b**	**Set4a**	**Set4b**	**Set5a**	**Set5b**	**Avg.**
TEPITOPE	0.776	0.740	0.740	0.754	0.763	0.750	0.651	0.661	0.729
PERUN	0.771	0.685	0.693	0.713	0.724	0.672	0.695	0.714	0.708
Gibbs Sampler^2^	0.803	0.775	0.75	0.762	0.793	0.787	0.621^1^	0.661^1^	0.744
LP_top2^2^	0.725	0.721	0.728	0.753	0.719	0.728	**0.815**^1^	**0.859**^1^	0.756
SKM	**0.870**	**0.832**	**0.823**	**0.821**	**0.862**	**0.827**	0.787	0.770	**0.824**

Comparing performance of SKM with results reported for the Gibbs Sampling method [27], "LP_top2" [21], and PERUN [7]. Best results shown in bold.

1: Best reported results, where Cysteines are treated as Alanines [27].

2: Best reported results of [21].

Results of the LP_top2 and Gibbs Sampler are from evaluation on the MHCBench sets. However, as is described in [21], training was performed on a training set consisting of selected samples from MHCPEP [1] and SYFPEITHI [53]. However, MHCBench mainly consists of samples from MHCPEP, and a large overlap exist between training and test sets (e.g. 502 of 646 samples of Set 4a).

The results on Table 4 show that the SKM is significantly better on 6 out of 8 benchmark sets (Set 1 to Set 4b). On sets 5a and 5b, another method, LP_top2, scored the best results (0.815 A_ROC _and 0.859 A_ROC_, respectively). The relative lower accuracies of the SKM method on these two data sets may be due to the small training set sizes (117 and 85); in training, the SKM method selected nearly all training samples as Support Vectors, indicating there may not be sufficient samples to properly describe the model space.

### Alleles from MHCPEP

All MHC Class II alleles from the MHCPEP database were evaluated. This database contains only binders, with binding strengths graded from low to high. An extensive number of alleles were tested, making it hard to obtain known non-binders for the sets. One approach is to use binders from other alleles as non-binders for the allele of interest. However, more than 10% of peptides were found to bind two or more alleles, which would generate a large amount of noise and uncertainty in predictions if used as non-binders. Instead, the non-binders were generated randomly in order to have the same length-distribution as the set of binders. Yewdell et al estimated that only one in 100 to 200 peptides will bind to an average allele [5], which makes this approach a reasonable approximation. Moreover, generating an equal amount of binders and non-binders creates a balanced data set well suited for computational experiments.

For each allele, the data set was extracted from MHCPEP and evaluated using 10-fold CV. As in previous experiments, a crude search for the best value of β was conducted. The results can be seen in Table 5. The SKM is able to model multiple MHC Class II alleles well, with an average A_ROC _of well over 0.9 in all cases except for HLA-DR10, which is a data set containing only 6 binders. Overall, the average performance (0.967 A_ROC_) is competitive (as good as or better) compared with literature results.

**Table 5:** Results of SKM on multiple MHC Class II alleles from MHCPEP

**MHC Allele**	**Species**	**#Samp**	**β**	**Acc.**	**Spec.**	**Sens.**	**A**_ROC_	**A**_ROC50_	**MCC**	**A**_OVER-ROC_
HLA-DR1^1^	Human	1346	0.04	0.9123	0.9153	0.9094	0.9712	0.8460	0.8247	0.0288
- *0101		474	0.06	0.8987	0.9114	0.8861	0.9673	0.8864	0.7977	0.0327
- *0102		12	0.005	0.8333	0.6667	1	0.9444	0.9444	0.7071	0.0556
HLA-DR2^1^	Human	648	0.15	0.9059	0.9692	0.8426	0.9608	0.8701	0.8183	0.0392
- *0201		44	0.8	0.8864	0.9091	0.8636	0.9360	0.9360	0.7735	0.0640
HLA-DR3^1^	Human	378	0.15	0.9101	0.9577	0.8624	0.9750	0.9216	0.8239	0.0250
- *0301		242	0.02	0.9339	0.9008	0.9669	0.9847	0.9676	0.8697	0.0153
HLA-DR4^1^	Human	1742	0.125	0.9248	0.9460	0.9036	0.9749	0.8677	0.8504	0.0251
- *0401		910	0.125	0.8890	0.9187	0.8593	0.9521	0.7989	0.7794	0.0479
- *0402		240	0.07	0.9	0.925	0.875	0.9717	0.9365	0.8010	0.0282
HLA-DR5	Human	398	0.125	0.9171	0.9799	0.8542	0.9717	0.9166	0.8408	0.0283
HLA-DR6	Human	46	0.25	0.9348	1	0.8696	0.9981	0.9981	0.8771	0.0019
HLA-DR7	Human	528	0.1	0.9034	0.9659	0.8409	0.9696	0.8965	0.8132	0.0304
HLA-DR8	Human	160	0.06	0.8938	0.8625	0.925	0.9683	0.9505	0.7890	0.0317
HLA-DR9	Human	192	0.2	0.9375	0.9896	0.8854	0.9779	0.9575	0.8798	0.0221
HLA-DR10	Human	12	5	0.5833	0.6667	0.5	0.6389	0.6389	0.1690	0.3611
HLA-DR11	Human	590	0.03	0.9169	0.9390	0.8949	0.9615	0.8847	0.8347	0.0385
HLA-DR14	Human	126	1	0.9762	1	0.9524	0.9934	0.9917	0.9535	0.0066
HLA-DR17	Human	308	0.03	0.9448	0.9545	0.9351	0.9802	0.9579	0.8898	0.0198
HLA-DR53	Human	72	0.2	0.8889	1	0.7778	0.9931	0.9931	0.7977	0.0069
HLA-DP9	Human	90	0.2	0.9889	0.9778	1	1	1	0.9780	0
HLA-DPw4	Human	38	0.01	0.7895	0.8421	0.7368	0.9058	0.9058	0.5822	0.0942
HLA-DQ1	Human	78	0.02	0.8974	0.9231	0.8718	0.9579	0.9579	0.7959	0.0420
HLA-DQ2	Human	210	0.08	0.8952	0.9714	0.8190	0.9664	0.936	0.7998	0.0336
HLA-DQ4	Human	194	0.2	0.8866	0.8969	0.8763	0.9557	0.9188	0.7734	0.0443

**Weighted average**		**363.12**	**0.120**	**0.9120**	**0.9390**	**0.8850**	**0.9687**	**0.8852**	**0.8263**	**0.0313**

Evaluating performance on multiple alleles using 10-fold CV. Average scores shown underneath, weighted by number of samples.

^1^All binders belonging to a group of alleles.

In [17], internal test sets of *0101 and *0301 extracted from MHCPEP were predicted with 0.91 A_ROC _and 0.88 A_ROC_, respectively. In comparison, the corresponding results for the SKM are 0.966 and 0.990. In [20], an iterative stepwise discriminant analysis was run on 400 HLA-DR1 high and moderate binders from MHCPEP and 743 non-binders. Classification accuracy using Jack-knife cross-validation was 91.4%. Here, the overall classification accuracy is slightly better at 92.2%.

In [16], a fuzzy neural network, combined with 3 amino acid property descriptors, was used to separate high, moderate and low binders from non-binders of HLA-DRB1*0401. Of the 321 binders and 312 non-binders collected for the allele, the highest performance was on strong (high affinity) binders vs. non-binders with an accuracy of 0.94 A_ROC_. However, for moderate and low affinity binders, their results were 0.93 and 0.88, respectively. For the SKM, the average A_ROC _value for *all *binders vs. non-binders is 0.952.

In [39], an ensemble classifier based on Support Vector Machines with a representation using QSAR descriptors achieved 0.917 A_ROC _on a data set consisting of 9-mers of HLA-DR4. As in this study, non-binders were generated randomly. The performance of the SKM (0.972 A_ROC_) on HLA-DR4 is significantly higher.

## Discussion

The proposed kernel method is shown to provide excellent discrimination between binders and non-binders for multiple alleles. It is able to model the dynamic MHC class II problem, and produce results that compare favourably with previously published results. The reason for the good performance may be due to several factors, and it is important to identify which of these are the most significant. The main focus of modelling was to consider the *full *length of peptides, as studies have shown that peptides outside the binding core can influence binding affinity [9]. Avoiding estimation of binding cores eliminates the potential for using faulty alignments, which can lead to increased model noise and, in turn, lower accuracy.

The use of similarity scores is a significant conceptual change in peptide evaluation, quantifying the overall similarity between peptides and interrelations between residues. This concept contrasts to the fixed-length representation (using binary positional system or amino acid properties) which enforces a direct pocket-to-pocket comparison of residues. Most static pattern recognition methods consider each input property to be a separate and independent entity, which is clearly not the case for a peptide string. Instead, higher order interactions within the peptide may also make a significant contribution to the modulation of affinity. Hattotuwagama et al. showed that the motif-dependence of Class II peptides is even weaker than that of class I epitopes [40]. Modelling such subtle effects, also seen in X-ray structures, are beyond the scope of much existing prediction technology. This change in concept could be a significant reason for the improved performance. Incorporating sub-optimal alignments into similarity scores have certainly contributed to an observed improvement, where low values of β produced the best performance.

Kernel methods, such as Support Vector Machines, have previously been shown to work well on biological problems, particularly when custom engineered kernels are used [31,33]. The SVM itself and the training principle of structural risk minimisation may have contributed to enhanced performance. However, simply applying SVMs to the MHC problem using aligned and truncated peptides (9-mers), in combination with a binary representation of amino acids similar to [18], did not produce promising results in initial experiments; custom kernels must be used to take full advantage of the kernel machines' excellent capacity for generalisation. Another advantage of using kernel methods is the ability to choose a kernel method independent of the choice of kernel itself. Thus, a kernel can readily be combined with a range of different kernel methods. This is useful when certain properties of the predictor are desired; e.g. some kernel methods can handle large-scale data sets while others allow for probabilistic interpretation of outputs.

Naturally, the proposed method is not without it's disadvantages. Firstly, the method is purely data-driven, in the sense that it relies solely on information derived from peptide data sets and thus does not consider MHC allele-specific structural information about the binding groove. While this may be seen as an advantage, since it keeps assumptions to a minimum, potentially important information is not considered, such as a specific pockets' preference for certain amino acids. Secondly, the method does not attempt to estimate alignments, which may be of interest, and finally, computational complexity and run-time speed could also be an issue for large scale testing. Calculating the kernel is time-consuming even with an efficient implementation; it cannot currently handle more than a few thousand samples before run-time becomes prohibitive.

Many potential improvements are possible that could either improve classification accuracy or provide more informative results. More advanced kernels could be developed: by increasing the importance of similarity scores of certain sized windows (e.g. length of 9) and subsequently weighting each residue in the window according to known binding motifs (e.g. [41]). This would have the advantage of incorporating allele-specific information into the method. Other improvements include modifying the kernel method to improve training or classification speeds, and developing new substitution matrices specific to the MHC domain similar to that undertaken for trans-membrane proteins [42,43]. Finally, the binary classification could be extended to a multi-class problem (separating non-binders from low, medium and high affinity binders), or directly predicting binding affinity by kernel regression, as Lui et al [44] has done for class I.

## Conclusion

The combination of a complex similarity score and an efficient kernel method are shown here to be a powerful tool for predicting MHC class II peptide binding affinity. The principle of using kernels to define similarities between sequences explicitly is a simple, yet flexible and powerful, way of modelling sequence data, and can readily be extended to address a variety of immunological and other biological problems.

## Methods

### Kernel engineering and string kernels

The mathematical formulation of kernel machines are described in details in books and publications (e.g. [30]). The *kernel function K *(*x*_*i*_, *x*) is the core of any kernel method, and can be used to incorporate a-priori knowledge of the problem into the model. A kernel function corresponds to a measurement of similarity or difference between any pair of samples (e.g. the *linear kernel *is a measure of the Euclidean distance between samples). However, kernel functions do not need to measure pair-wise similarities through a dot-product of vector representations. Instead, *explicit *measures of similarities between samples can be used; such as similarities between amino acid strings: *string kernels*. This enables the full length of each peptide to be incorporated into the model, including information known to be hidden in flanking residues [10-14,45].

### Local alignment kernels

The principle of local alignments has been shown to provide a powerful approach to detecting relationships between sequences, using the optimal local alignment via the Smith-Waterman algorithm [46] and it's efficient (PSI-)BLAST approximations [47]. Therefore, we utilise a complex kernel comprising several sub-kernels based on the Smith-Waterman algorithm [32]. On a protein homology detection problem, this approach was found to significantly outperform scores based solely on optimal alignments [31].

Local Alignment Kernels are *convolution kernels *[48] consisting of a number of simple sub-kernels:

K1⋅K2⋅...⋅Kp(x,y)=∑x=x1...xp,y=y1...ypK1(x1,y1)⋅...⋅Kp(xp,yp)
 MathType@MTEF@5@5@+=feaafiart1ev1aaatCvAUfKttLearuWrP9MDH5MBPbIqV92AaeXatLxBI9gBaebbnrfifHhDYfgasaacH8akY=wiFfYdH8Gipec8Eeeu0xXdbba9frFj0=OqFfea0dXdd9vqai=hGuQ8kuc9pgc9s8qqaq=dirpe0xb9q8qiLsFr0=vr0=vr0dc8meaabaqaciaacaGaaeqabaqabeGadaaakeaafaqaaeGabaaabaGaem4saS0aaSbaaSqaaiabigdaXaqabaGccqGHflY1cqWGlbWsdaWgaaWcbaGaeGOmaidabeaakiabgwSixlabc6caUiabc6caUiabc6caUiabgwSixlabdUealnaaBaaaleaacqWGWbaCaeqaaOGaeiikaGIaemiEaGNaeiilaWIaemyEaKNaeiykaKIaeyypa0dabaWaaabuaeaacqWGlbWsdaWgaaWcbaGaeGymaedabeaakiabcIcaOiabdIha4naaBaaaleaacqaIXaqmaeqaaOGaeiilaWIaemyEaK3aaSbaaSqaaiabigdaXaqabaGccqGGPaqkcqGHflY1cqGGUaGlcqGGUaGlcqGGUaGlcqGHflY1cqWGlbWsdaWgaaWcbaGaemiCaahabeaakiabcIcaOiabdIha4naaBaaaleaacqWGWbaCaeqaaOGaeiilaWIaemyEaK3aaSbaaSqaaiabdchaWbqabaGccqGGPaqkaSqaaiabdIha4jabg2da9iabdIha4naaBaaameaacqaIXaqmaeqaaSGaeiOla4IaeiOla4IaeiOla4IaemiEaG3aaSbaaWqaaiabdchaWbqabaWccqGGSaalcqWG5bqEcqGH9aqpcqWG5bqEdaWgaaadbaGaeGymaedabeaaliabc6caUiabc6caUiabc6caUiabdMha5naaBaaameaacqWGWbaCaeqaaaWcbeqdcqGHris5aaaaaaa@79C6@

Eq. 0.1

Where the components K_1_...K_p _consists of three different kernels: (a) A constant kernel K_const_, (b) a kernel for measuring the difference between aligned letters K_align_, and (c) a kernel for penalizing gaps K_gap_:

Kconst(x,y)=1Kalign(x,y)={0,if|x|≠1 or|y|≠1eβ∗S(x,y)otherwiseKgap(x,y)=eβ(g(|x|)+g(|y|))
 MathType@MTEF@5@5@+=feaafiart1ev1aaatCvAUfKttLearuWrP9MDH5MBPbIqV92AaeXatLxBI9gBaebbnrfifHhDYfgasaacH8akY=wiFfYdH8Gipec8Eeeu0xXdbba9frFj0=OqFfea0dXdd9vqai=hGuQ8kuc9pgc9s8qqaq=dirpe0xb9q8qiLsFr0=vr0=vr0dc8meaabaqaciaacaGaaeqabaqabeGadaaakeaafaqaaeWabaaabaGaem4saS0aaSbaaSqaaiabdogaJjabd+gaVjabd6gaUjabdohaZjabdsha0bqabaGccqGGOaakcqWG4baEcqGGSaalcqWG5bqEcqGGPaqkcqGH9aqpcqaIXaqmaeaacqWGlbWsdaWgaaWcbaGaemyyaeMaemiBaWMaemyAaKMaem4zaCMaemOBa4gabeaakiabcIcaOiabdIha4jabcYcaSiabdMha5jabcMcaPiabg2da9maaceqabaqbaeqabiGaaaqaaiabicdaWaqaaiabcYcaSiabdMgaPjabdAgaMjabcYha8jabdIha4jabcYha8jabgcMi5kabigdaXiabbccaGiabd+gaVjabdkhaYjabcYha8jabdMha5jabcYha8jabgcMi5kabigdaXaqaaiabdwgaLnaaCaaaleqabaacciGae8NSdiMaey4fIOIaem4uamLaeiikaGIaemiEaGNaeiilaWIaemyEaKNaeiykaKcaaaGcbaGaem4Ba8MaemiDaqNaemiAaGMaemyzauMaemOCaiNaem4DaCNaemyAaKMaem4CamNaemyzaugaaaGaay5EaaaabaGaem4saS0aaSbaaSqaaiabdEgaNjabdggaHjabdchaWbqabaGccqGGOaakcqWG4baEcqGGSaalcqWG5bqEcqGGPaqkcqGH9aqpcqWGLbqzdaahaaWcbeqaaiab=j7aIjabcIcaOiabdEgaNjabcIcaOiabcYha8jabdIha4jabcYha8jabcMcaPiabgUcaRiabdEgaNjabcIcaOiabcYha8jabdMha5jabcYha8jabcMcaPiabcMcaPaaaaaaaaa@9ACD@

Eq. 0.2

where x and y are the amino acid sequences, *S *(*x*, *y*) the Smith-Waterman score, *g *(·) the gap penalty function, and β a scaling parameter to adjust importance of gaps and sub-optimal alignments.

The Smith-Waterman score *SW*_*S,g*(π) _is calculated as:

SS,g(π)=∑i=1|π|S(xπ1(i),yπ2(i))−∑i=1|π|−1[g(π1(i+1)−π1(i))+g(π2(i+1)−π2(i))]SWS,g(π)(x,y)=max⁡π∈Π(x,y)SS,g(π)
 MathType@MTEF@5@5@+=feaafiart1ev1aaatCvAUfKttLearuWrP9MDH5MBPbIqV92AaeXatLxBI9gBaebbnrfifHhDYfgasaacH8akY=wiFfYdH8Gipec8Eeeu0xXdbba9frFj0=OqFfea0dXdd9vqai=hGuQ8kuc9pgc9s8qqaq=dirpe0xb9q8qiLsFr0=vr0=vr0dc8meaabaqaciaacaGaaeqabaqabeGadaaakeaafaqaaeGabaaabaGaem4uam1aaSbaaSqaaiabdofatjabcYcaSiabdEgaNjabcIcaOGGaciab=b8aWjabcMcaPaqabaGccqGH9aqpdaaeWbqaaiabdofatnaabmaabaGaemiEaG3aaSbaaSqaaiab=b8aWjabigdaXiabcIcaOiabdMgaPjabcMcaPaqabaGccqGGSaalcqWG5bqEdaWgaaWcbaGae8hWdaNaeGOmaiJaeiikaGIaemyAaKMaeiykaKcabeaaaOGaayjkaiaawMcaaaWcbaGaemyAaKMaeyypa0JaeGymaedabaGaeiiFaWNae8hWdaNaeiiFaWhaniabggHiLdGccqGHsisldaaeWbqaaiabcUfaBjabdEgaNjabcIcaOiab=b8aWnaaBaaaleaacqaIXaqmaeqaaOGaeiikaGIaemyAaKMaey4kaSIaeGymaeJaeiykaKIaeyOeI0Iae8hWda3aaSbaaSqaaiabigdaXaqabaGccqGGOaakcqWGPbqAcqGGPaqkcqGGPaqkcqGHRaWkcqWGNbWzcqGGOaakcqWFapaCdaWgaaWcbaGaeGOmaidabeaakiabcIcaOiabdMgaPjabgUcaRiabigdaXiabcMcaPiabgkHiTiab=b8aWnaaBaaaleaacqaIYaGmaeqaaOGaeiikaGIaemyAaKMaeiykaKIaeiykaKIaeiyxa0faleaacqWGPbqAcqGH9aqpcqaIXaqmaeaacqGG8baFcqWFapaCcqGG8baFcqGHsislcqaIXaqma0GaeyyeIuoaaOqaaGqaciab+nfatjab+DfaxnaaBaaaleaacqGFtbWucqGFSaalcqGFNbWzieaacqqFOaakcqWFapaCcqqFPaqkaeqaaOGaeiikaGIaemiEaGNaeiilaWIaemyEaKNaeiykaKIaeyypa0ZaaCbeaeaacyGGTbqBcqGGHbqycqGG4baEaSqaaiab=b8aWjabgIGiolabfc6aqjabcIcaOiabdIha4jabcYcaSiabdMha5jabcMcaPaqabaGccqWGtbWudaWgaaWcbaGaem4uamLaeiilaWIaem4zaCMaeiikaGIae8hWdaNaeiykaKcabeaaaaaaaa@AD37@

Eq. 0.3

Where *π *is the alignment between two sequences *x *and *y*, *S *(·) is a substitution matrix and *g *(·) a gap penalty function.

The component sub-kernels are combined by convolution to represent a kernel for an alignment of length *n*. The Local Alignment score is the sum over all possible alignments in the sequence:

K(n)(x,y)=Kconst⋅(Kalign⋅Kgap)(n−1)⋅Kalign⋅KconstKLA(x,y)=∑i=0NKi(x,y)
 MathType@MTEF@5@5@+=feaafiart1ev1aaatCvAUfKttLearuWrP9MDH5MBPbIqV92AaeXatLxBI9gBaebbnrfifHhDYfgasaacH8akY=wiFfYdH8Gipec8Eeeu0xXdbba9frFj0=OqFfea0dXdd9vqai=hGuQ8kuc9pgc9s8qqaq=dirpe0xb9q8qiLsFr0=vr0=vr0dc8meaabaqaciaacaGaaeqabaqabeGadaaakeaafaqaaeGabaaabaGaem4saS0aaSbaaSqaaiabcIcaOiabd6gaUjabcMcaPaqabaGccqGGOaakcqWG4baEcqGGSaalcqWG5bqEcqGGPaqkcqGH9aqpcqWGlbWsdaWgaaWcbaGaem4yamMaem4Ba8MaemOBa4Maem4CamNaemiDaqhabeaakiabgwSixlabcIcaOiabdUealnaaBaaaleaacqWGHbqycqWGSbaBcqWGPbqAcqWGNbWzcqWGUbGBaeqaaOGaeyyXICTaem4saS0aaSbaaSqaaiabdEgaNjabdggaHjabdchaWbqabaGccqGGPaqkdaahaaWcbeqaaiabcIcaOiabd6gaUjabgkHiTiabigdaXiabcMcaPaaakiabgwSixlabdUealnaaBaaaleaacqWGHbqycqWGSbaBcqWGPbqAcqWGNbWzcqWGUbGBaeqaaOGaeyyXICTaem4saS0aaSbaaSqaaiabdogaJjabd+gaVjabd6gaUjabdohaZjabdsha0bqabaaakeaacqWGlbWsdaWgaaWcbaGaemitaWKaemyqaeeabeaakiabcIcaOiabdIha4jabcYcaSiabdMha5jabcMcaPiabg2da9maaqahabaGaem4saS0aaSbaaSqaaiabdMgaPbqabaGccqGGOaakcqWG4baEcqGGSaalcqWG5bqEcqGGPaqkaSqaaiabdMgaPjabg2da9iabicdaWaqaaiabd6eaobqdcqGHris5aaaaaaa@86DE@

Eq. 0.4

where *N *is the number of possible alignments.

The above formulation results in a computational complexity exponential with |*x*| and |*y*|, and is thus not a feasible solution for this problem. Hence, a dynamic programming algorithm by [32] is used, which is a slight modification of the Smith-Waterman algorithm [46]. The kernel computation is done in *O *(*n*^2^·|*x*|·|*y*|), where *n *is the number of samples, and |*x*| and |*y*| are the lengths of the peptide strings. In the context of MHC-peptide binding, the gap penalty term must be maximised since gaps are not possible within bound peptides.

Calculating the sub-kernels requires the following two parameters: the substitution matrix *S *(·) in Eq. 0.3, and the β parameter in Eq. 0.2. *S *(·) quantifies a similarity between pairs of amino acids, and is a well-known term in bioinformatics with numerous substitution matrices designed from evolutionary, physicochemical or structural properties (a list can be found at [23]). The β parameter regulates the effect of individual contributions from alignments, and allows adjustment of the relative importance between low- and high scoring alignments. When β is low, the model will increase the importance of low scoring (sub-optimal) alignments to quantify the similarity between sequences. Similarly, as *β *→ ∞ the contributions from sub-optimal values is reduced.

All kernels must be symmetric and positive semi-definite. Some values of β and substitution matrices S in Eq. 0.2 resulted in invalid kernels, and caused convergence problems. A trick used in [32] subtracts the smallest negative Eigenvalue from the diagonal of the kernel to ensure kernels are positive semi-definite. Symmetry of the resulting kernel, *K*_*LA*_, is guaranteed as long as substitution matrix *S *(·) is symmetric.

### Data set

Multiple data sets were used in the experiments. Eight benchmark data sets with samples of known binders and non-binders of the HLA-DRB1*0401 allele were taken from MHCBench [3]. Within the data sets, peptide strings are assigned binding strengths of level 0 (non-binders) to 4 (strong binders), and are collected from multiple sources, mainly MHCPEP [1]. The sets are derived from the same base set of peptides, created with varying levels of curation. Set 1 includes all peptides whereas Set5b is a homology reduced set containing only natural peptides. Data set sizes range from 85 to 1017 samples with peptide lengths of 9 to 33. As these sets have been used in many published experiments [21,26,27], we use them in preference to alternatives.

In addition to the 8 sets, two data sets from specific alleles HLA-DRB1*0101 and HLA-DRB1*0301 were taken from MHCBN [2]. The data sets separate peptides into binders (low, moderate, and high), and non-binders (peptides having IC_50 _values of more than 50,000 nm). Finally, binders from multiple alleles from the MHCPEP database [1] were used. In the database, peptides are labelled as having low, moderate or high binding affinity.

### Experimental setup

MATLAB [49] was used as the testing environment, with assistance of the SPIDER toolbox [50]. The SKM was calculated with a C++ Mex implementation based on [32]. Testing machine was an Intel Pentium M 1.4GHz.

In all experiments, samples were randomly permuted and subsequently evaluated using N-fold cross validation (CV). Targets y_i _were divided into binders and non-binders; y_i _∈ {-1, 1}. For each left-out fold, a model was trained on the remaining folds to separate samples into binders and non-binders. The trained model was then evaluated on the left out fold. In addition, a rough search for good values of β was performed by performing a full CV test for each value of β (typically evaluating 10–15 different values of β). CV is well-suited for model assessment of small data sets. However, some studies report the possibility of high variance in results using CV (e.g. [51]).

For assessing performance, several measures were used: Overall prediction accuracy, sensitivity, specificity, Matthew's Correlation Coefficient (MCC), area under receiver operating characteristic curve (A_ROC_), and the A_ROC _score up to the first 50 false positives (A_ROC50_). Finally, in cases where A_ROC _scores were close to 1.0 (perfect classification), the error term of area *over *the ROC curve, A_OVER_ROC_, was used as well.

For experiments, data sets were curated by removing duplicates as well as unnatural peptides with more than 75% Alanine [21,27]. An overview of the data sets can be seen in Table 1.

## Authors' contributions

JS conducted the data mining and modelling. DRF conceived, designed, oversaw, and interpreted the study. JS and DRF jointly drafted the manuscript. All authors read and approved the final manuscript.
